# A global analysis of gene expression in *Fibrobacter succinogenes* S85 grown on cellulose and soluble sugars at different growth rates

**DOI:** 10.1186/s13068-018-1290-x

**Published:** 2018-10-27

**Authors:** Anthony P. Neumann, Paul J. Weimer, Garret Suen

**Affiliations:** 1 Department of Bacteriology, University of Wisconsin-Madison, Madison, WI USA; 2 Agricultural Research Service, United States Department of Agriculture, Madison, WI USA

**Keywords:** *Fibrobacter succinogenes* S85, Cellulose, Plant cell wall polysaccharide, RNA-Seq, Gene expression, Cellulase, Hemicellulase

## Abstract

**Background:**

Cellulose is the most abundant biological polymer on earth, making it an attractive substrate for the production of next-generation biofuels and commodity chemicals. However, the economics of cellulose utilization are currently unfavorable due to a lack of efficient methods for its hydrolysis. *Fibrobacter succinogenes* strain S85, originally isolated from the bovine rumen, is among the most actively cellulolytic mesophilic bacteria known, producing succinate as its major fermentation product. In this study, we examined the transcriptome of *F. succinogenes* S85 grown in continuous culture at several dilution rates on cellulose, cellobiose, or glucose to gain a system-level understanding of cellulose degradation by this bacterium.

**Results:**

Several patterns of gene expression were observed for the major cellulases produced by *F. succinogenes* S85. A large proportion of cellulase genes were constitutively expressed, including the gene encoding for Cel51A, the major cellulose-binding endoglucanase produced by this bacterium. Moreover, other cellulase genes displayed elevated expression during growth on cellulose relative to growth on soluble sugars. Growth rate had a strong effect on global gene expression, particularly with regard to genes predicted to encode carbohydrate-binding modules and glycoside hydrolases implicated in hemicellulose degradation. Expression of hemicellulase genes was tightly regulated, with these genes displaying elevated expression only during slow growth on soluble sugars. Clear differences in gene expression were also observed between adherent and planktonic populations within continuous cultures growing on cellulose.

**Conclusions:**

This work emphasizes the complexity of the fiber-degrading system utilized by *F. succinogenes* S85, and reinforces the complementary role of hemicellulases for accessing cellulose by these bacteria. We report for the first time evidence of global differences in gene expression between adherent and planktonic populations of an anaerobic bacterium growing on cellulose at steady state during continuous cultivation. Finally, our results also highlight the importance of controlling for growth rate in investigations of gene expression.

**Electronic supplementary material:**

The online version of this article (10.1186/s13068-018-1290-x) contains supplementary material, which is available to authorized users.

## Background

Cellulose is the major structural polysaccharide in plant cell walls and is estimated to be the most abundant biological polymer on earth [1]. As a result, cellulose is of fundamental importance to herbivorous animals, as well as an attractive substrate for the production of next-generation renewable fuels and commodity chemicals. However, the exploitation of cellulose as a source for energy is severely limited by the chemical nature of the cellulose molecule itself and the complex architecture of the plant cell wall. Cellulose is composed of repeating units of cellobiose, the β-(1-4)-linked disaccharide of glucose, arranged into linear chains that are further assembled into higher-order structures of increasing complexity including planar sheets, crystals, microfibrils, and fibers [2]. Hydrogen bonding among adjacent chains and sheets reinforces the fiber, which is impermeable to water. In the plant cell wall, cellulose fibers are typically embedded in a matrix of hemicellulose, pectin, and lignin, further adding to their stability [3]. Nevertheless, microorganisms have evolved mechanisms to access and depolymerize cellulose to utilize its constituent sugars for growth [4]. Some of the most prolific of these cellulose-degrading microbes live in the herbivore gut [5].

Ruminants, animals with an expanded foregut for microbial fermentation of plant matter, are among the most successful herbivores, and are of economic importance to humans throughout the world. The rumen microbiota has been extensively studied because of its superior cellulolytic ability and vital connection to the health and well-being of its host [6]. Species of bacteria in the genera *Ruminococcus* and *Fibrobacter* are typically the most abundant cellulose-degrading microbes in the rumen, and are among the most cellulolytic organisms identified to date [7]. *Fibrobacter succinogenes*, the major *Fibrobacter* species in the rumen, is especially adept at solubilizing highly ordered forms of cellulose [8, 9]. Moreover, strains of *F. succinogenes* have demonstrated a greater ability to digest cellulose from intact forages than other species of fibrolytic rumen bacteria [10]. The *F. succinogenes* type strain, S85, was originally isolated from a bovine rumen fluid sample and has been utilized extensively as a model to study *Fibrobacter* physiology, as well as their unique mechanism of polysaccharide degradation [11–14]. In addition to being one of the most important members of the ruminal microbial community, *F. succinogenes* has attracted interest because it is one of the few known cellulolytic microorganisms to produce the valuable chemical succinic acid as its major fermentation product [15, 16]. Although its specific productivity and endpoint succinate concentrations are far lower than those of starch- or sugar-fermenting succinate producers, due in part to the narrow pH tolerance range of *F. succinogenes* and growth rate limitations imposed by accessible surface area of its cellulosic substrates, S85 has shown industrial potential in co-culture with *Clostridium kluyveri* for the conversion of cellulosic substrates into caproic acid using the carboxylate platform [17].

Much progress has been made in our understanding of cellulolysis by *Fibrobacter*, but many important details of the process remain poorly understood. Like other anaerobic cellulose-degrading bacteria, *Fibrobacter* requires close physical contact with the substrate to achieve efficient hydrolysis and growth [18–20]. However, unlike many clostridia, the well-studied group of anaerobic cellulose-degrading bacteria, *Fibrobacter,* does not produce cellulosomes to facilitate the process [21, 22]. A Gram-negative cell envelope also differentiates *Fibrobacter* from the cellulolytic clostridia, and several unique outer membrane (OM) proteins have been implicated in cellulose-binding including fibro-slime domain containing proteins and a type IV pilin [23]. The OM is apparently only loosely associated with the cell, and vesicles exhibiting hydrolytic activity have been observed originating from the OM [24, 25]. The *F. succinogenes* S85 genome has been predicted to encode 104 different glycoside hydrolases (GHs), representing 3.37% of all S85 genes, which is among the highest percentage of GHs in any bacterial species [26]. Several of these enzymes have been purified and characterized, but they generally have limited or no activity against crystalline cellulose [27, 28]. *F. succinogenes* S85 has demonstrated the ability to readily solubilize a variety of hemicelluloses and pectins from intact plant cell walls, but available evidence indicates that this bacterium only utilizes hexoses for growth, particularly those arising from the breakdown of cellulose [22].

Although many proteins have been implicated in the process of cellulose degradation by *F. succinogenes* S85, it is not well known how they interact or how their expression may be regulated. Some reports have proposed constitutive expression of cellulases by S85, while others have suggested tight regulation [29, 30]. The goal of this work was to better understand the mechanism of cellulose degradation by strain S85 through genome-wide analysis of gene expression using RNA sequencing. To determine the effect of carbon source on gene expression, S85 was cultured on cellulose, cellobiose, or glucose at several growth rates. Cells from cellulose-grown cultures were fractionated into cellulose-adherent and cellulose-planktonic populations and evaluated separately. The effect of growth rate was controlled by analyzing populations at steady state during continuous culture. We hypothesized that genes important for digesting cellulose would be upregulated during growth on cellulose compared to growth on soluble sugars, and that patterns of co-expression would allow us to identify cellular processes that act synergistically in cellulose hydrolysis. Using this approach, we obtained evidence for tight control over the expression of hemicellulases by S85, in addition to identifying a subset of cellulases that were highly expressed under all conditions examined, while a separate group of cellulases showed elevated expression during growth on cellulose. This work also confirmed the heterogeneous nature of bacterial populations cultured on an insoluble substrate, and reinforced the importance of controlling for the effect of growth rate when investigating microbial gene expression.

## Results

### General characteristics of *F. succinogenes* S85 continuous cultures

Summary statistics for the culture conditions in this study, and the results of statistical analysis of fermentation products in steady-state culture supernatants are reported in Table 1. Dilution rates calculated at the time of sampling were all close to their intended targets. Succinate, detected at concentrations ranging from 9.60 to 15.56 mM, was the major fermentation product regardless of substrate type or growth rate. Acetate was the only other fermentation product detected at concentrations > 1 mM. Traces of formate (< 1 mM) were detected in most cultures, but the concentrations detected, near the method’s detection limit, were too low for reliable statistical analyses. Linear regression followed by ANOVA identified a significant interaction between carbon source and growth rate for both succinate (*P* = 1.55 × 10^−3^) and acetate (*P* = 1.18 × 10^−6^) production. Concentrations of both fermentation products fell much faster with increasing growth rate during growth on cellulose, compared with growth on glucose or cellobiose; this was expected because cellulose utilization varied considerably with dilution rate, while cellobiose and glucose consumption was nearly complete at all dilution rates. In cellulose-fed continuous cultures, total cellulose consumption (1.828 g L^−1^) was highest in cultures growing at the lowest growth rate (*D* = 0.018 h^−1^), and cell protein concentration followed the pattern cellulose > cellobiose > glucose (Additional file 1: Table S1), as expected from the known energetic savings of an intracellular cellodextrin phosphorylase enzyme system [4]. In cellulose-grown cultures, almost all cellulose particles were fully colonized by cells, and the vast majority of the cells were adherent to cellulose (89.9%, 95.4% and 96.0% for *D* = 0.018, 0.047 and 0.068 h^−1^, respectively, based on protein analysis of adherent and non-adherent cell pellets; Additional file 1: Table S1). These results are consistent with previous demonstrations that cellulose degradation by this organism follows first-order kinetics whose rate is limited by the gross specific surface area of the cellulose particles [31].

**Table 1:** Culture summary statistics

Substrate	Target D (h^−1^)	Actual D (h^−1^)	Succinate	Acetate	Formate	Total soluble sugar	SC20 consumed (g L^−1^)
Cellulose	0.018	0.018	15.56 ± 2.40	7.24 ± 1.00	0.52 ± 0.39	1.31 ± 0.08	1.83
Cellulose	0.047	0.047	7.05 ± 0.97	3.11 ± 0.52	0.21 ± 0.38	0.56 ± 0.06	0.31
Cellulose	0.068	0.068	9.56 ± 1.14	2.92 ± 0.47	0.09 ± 0.15	0.72 ± 0.05	0.74
Glucose	0.018	0.019	12.70 ± 0.41	5.01 ± 0.15	0.17 ± 0.03	0.81 ± 0.04	
Glucose	0.047	0.047	10.09 ± 0.49	4.39 ± 1.40	0.69 ± 0.46	1.45 ± 0.14	
Glucose	0.068	0.067	10.80 ± 0.35	3.86 ± 0.15	0.31 ± 0.08	0.98 ± 0.14	
Glucose	0.200	0.198	11.25 ± 1.68	3.79 ± 1.90	0.94 ± 0.55	0.84 ± 0.03	
Cellobiose	0.018	0.016	13.33 ± 0.99	4.66 ± 0.36	ND	0.97 ± 0.05	
Cellobiose	0.047	0.047	10.54 ± 0.44	3.36 ± 0.13	0.21 ± 0.11	1.19 ± 0.05	
Cellobiose	0.068	0.068	10.69 ± 0.36	3.58 ± 0.08	0.44 ± 0.09	1.01 ± 0.17	
Cellobiose	0.200	0.193	10.03 ± 0.49	3.32 ± 0.16	0.50 ± 0.08	0.82 ± 0.07	
ANOVA*		Growth rate (GR)	0.070	0.007			
		Substrate (S)	0.518	0.095			
		GR × S	0.002	1.18 × 10^−6^			

Values of fermentation products and total soluble sugars are mean ± 1 standard deviation of three biological replicates

*D* dilution rate, *ND* not detected

*Results reported for ANOVA of the two factors and interaction are *P* values

Regardless of substrate fed, all cultures contained 0.8–1.4 mM of total soluble sugars. These soluble sugar concentrations averaged 0.86 ± 0.40, 1.02 ± 0.29, and 1.00 ± 0.15 mM (mean ± S.D.) for cellulose, glucose, and cellobiose, respectively (means not different, *P* > 0.05). Separate analysis of reducing sugars indicated that the average degree of polymerization of the soluble sugars (calculated as mM total sugars/mM reducing sugars) averaged 1.41 across all substrates and did not differ across substrate.

### Genome-wide expression by *F. succinogenes* S85 across conditions

**Fig. 1:**
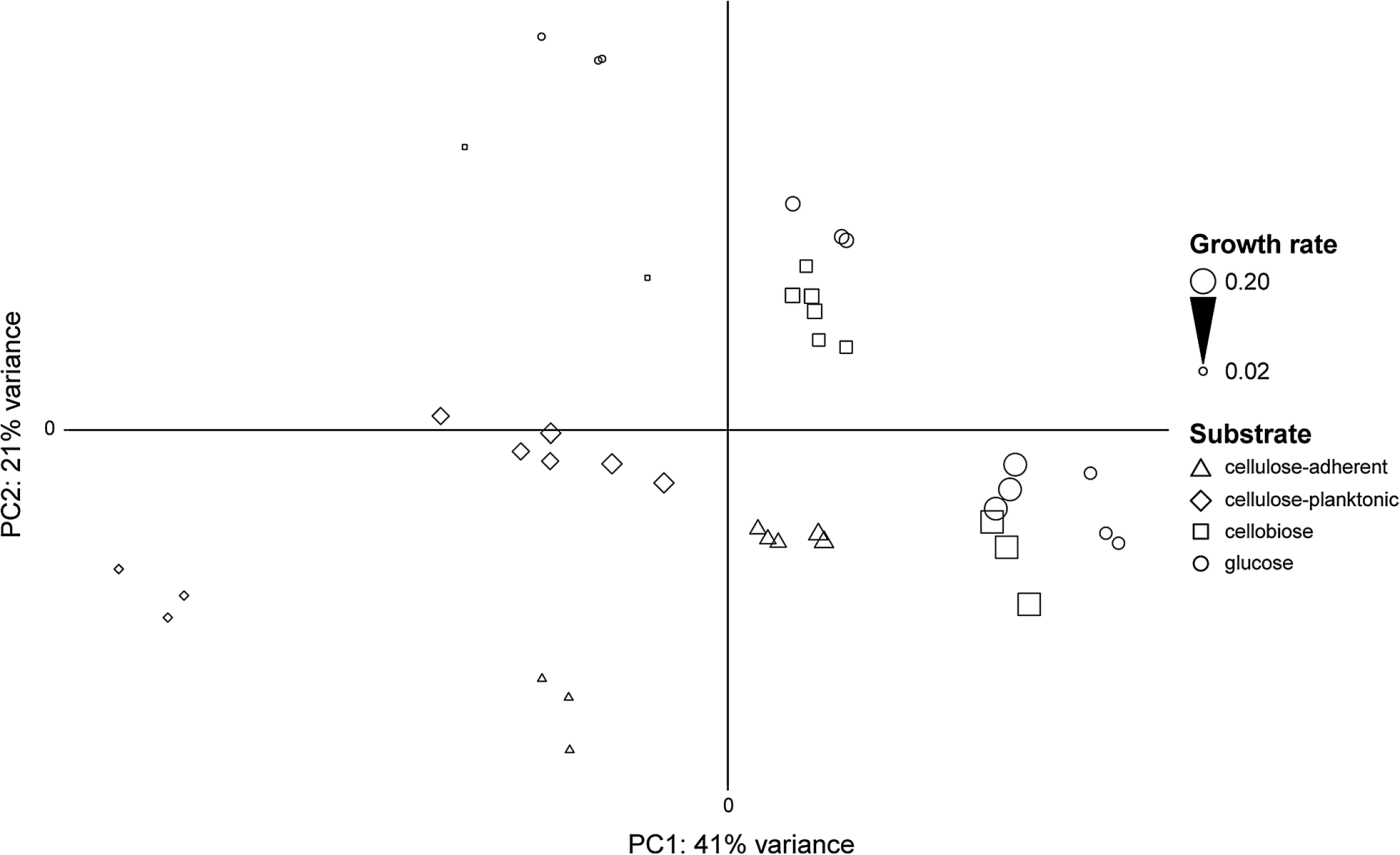
Principal components analysis of transcriptomes. The scatter plot shows the transcriptomes plotted according to the first two dimensions of principal components analysis. Cellulose-adherent samples are plotted as open triangles, cellulose-planktonic samples are plotted as open diamonds, cellobiose samples are plotted as open squares, and glucose samples are plotted as open circles. The sizes of the shapes increase as a function of growth rate. The plot shows the results of each substrate-growth rate combination performed in triplicate with the exception of cellobiose at the lowest growth rate (see “Methods”)

**Fig. 2:**
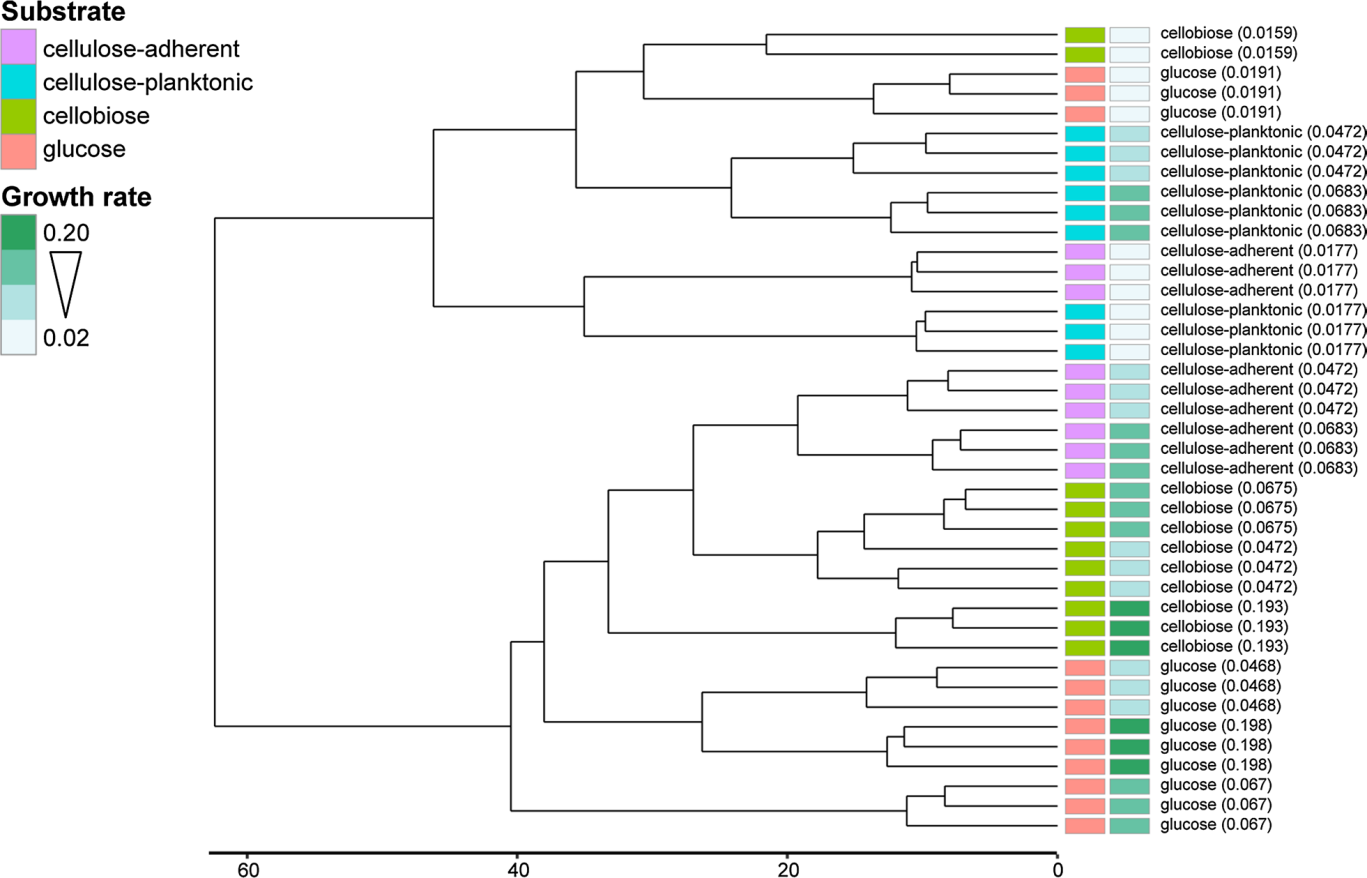
Hierarchical clustering of transcriptomes. The dendrogram shows the results of complete-linkage hierarchical clustering of the transcriptomes based on Euclidean distance. Samples are colored according to substrate as follows: purple = cellulose-adherent, blue = cellulose-planktonic, green = cellobiose, red = glucose. Samples are separately colored according to growth rate from light (slow) to dark green (fast). Samples are labeled according to substrate type followed by empirically determined growth rates in parentheses

Figure 1 shows the relationships among the transcriptomes of all samples according to the first two principal components of PCA. The first principal component (PC1) explained 41% of the total variance and generally differentiated the samples according to growth rate, with faster growing populations tending to have higher values of PC1 compared to those growing more slowly on the same substrate. PC1 also clearly differentiated the two subpopulations of cellulose cultures. Cellulose-adherent populations had higher values of PC1 compared to the corresponding cellulose-planktonic populations recovered from the same continuous culture. The second principal component (PC2) explained 21% of the total variance and generally separated samples by substrate. Populations growing on glucose tended to have the highest values of PC2, which were slightly higher than those of populations growing on cellobiose. Values of PC2 for both cellulose-adherent and cellulose-planktonic populations were lower than those of populations growing on soluble sugars, but were lowest at the lowest dilution rate. Figure 2 shows a dendrogram resulting from hierarchical clustering of the sample expression profiles. Biological replicates generally clustered together in the dendrogram, indicating good reproducibility within a condition at a given growth rate. Two major clusters were identified that segregated primarily by growth rate, with the exception of the cellulose-planktonic samples, which all clustered, regardless of growth rate, with only the slowest growing populations from the other conditions. Within the two major clusters, the samples grouped primarily according to substrate type.

### General patterns of gene expression by *F. succinogenes* S85

A total of 3119 genes were identified in the S85 transcriptome (Additional file 1: Table S2). Among these, 639 genes (20.49%) were significantly affected by growth rate (false discovery rate (FDR) < 0.01 and log2 fold change (LFC) > 1.5) (Additional file 1: Table S3), and 483 genes (15.49%) were significantly affected by carbon substrate, FDR < 0.01 and LFC > 1.5 (Additional file 1: Table S4). Evidence of an effect on expression by both growth rate and substrate was observed for 215 genes (6.89%). Hierarchical clustering of all genes identified 17 distinct clusters of genes that grouped together according to similar expression profiles across the different growth rates and carbon substrates (Table 2, Additional file 1: Table S2). Most genes in the S85 transcriptome (66.56%) were placed in gene clusters 1–3, the 3 largest gene clusters identified (Table 2). No evidence for a systematic effect of growth rate or substrate type was observed for gene clusters 1–3; however, certain functional categories of genes were enriched in these clusters compared to the full S85 transcriptome: cluster 1 was enriched for genes in the KEGG category: bacterial motility proteins; cluster 2 was enriched for peptidoglycan biosynthesis and polyketide sugar unit biosynthesis; and gene cluster 3 was enriched for the KEGG categories amino acid related enzymes, aminoacyl-tRNA biosynthesis, biotin metabolism, porphyrin and chlorophyll metabolism, ribosome, transfer RNA biogenesis, and translation factors. Three genes predicted to be involved in glycogen metabolism were also identified among these genes, consisting of two glycogen synthases in cluster 1 (Fisuc_1515 and Fisuc_2067) and a glycogen phosphorylase in cluster 3 (Fisuc_2097), that were highly expressed, independent of growth rate or substrate type (Additional file 1: Table S2). This result is not surprising, given that glycogen turnover has been reported to be important in the overall carbon flux in *F. succinogenes* during growth on both cellulose and soluble sugars [14, 32]. The remaining 14 gene clusters contained a maximum of 167 and a minimum of 21 genes. Most of these clusters, 10 out of 14 (71.43%), were enriched in genes affected by growth rate, substrate, or both growth rate and substrate (Table 2).

**Table 2:** *P*-values of significant enrichments among clusters of co-regulated genes

Cluster	# of genes	Growth rate	Substrate	KEGG category		CAZy category	
1	1157	NS	NS	Bacterial motility proteins	8.37 × 10^−3^	NS	
2	507	NS	NS	Peptidoglycan biosynthesis	2.12 × 10^−3^	CBM51	4.70 × 10^−2^
				Polyketide sugar unit biosynthesis	4.80 × 10^−2^		
3	412	NS	NS	Amino acid related enzymes	1.06 × 10^−2^	NS	
				Aminoacyl-tRNA biosynthesis	1.63 × 10^−2^		
				Biotin metabolism	6.17 × 10^−3^		
				Porphyrin and chlorophyll metabolism	2.16 × 10^−3^		
				Ribosome	1.29 × 10^−14^		
				Transfer RNA biogenesis	1.64 × 10^−6^		
				Translation factors	3.36 × 10^−2^		
4	167	2.70 × 10^−6^	4.80 × 10^−19^	NS		NS	
5	164	NS	NS	Ribosome	4.36 × 10^−9^	NS	
6	140	NS	NS	NS		NS	
7	92	6.39 × 10^−04^	2.11 × 10^−48^	Chaperones and folding catalysts	1.64 × 10^−2^	NS	
				Prokaryotic defense system	3.51 × 10^−3^		
8	81	NS	NS	Phenylalanine, tyrosine and tryptophan biosynthesis	2.56 × 10^−2^	GT4	1.72 × 10^−3^
9	78	2.83 × 10^−27^	3.48 × 10^−16^	ABC transporters	2.24 × 10^−3^	NS	
				Chloroalkane and chloroalkene degradation	2.35 × 10^−8^		
				Cysteine and methionine metabolism	2.56 × 10^−7^		
				Nitrogen metabolism	2.48 × 10^−6^		
				Quorum sensing	9.65 × 10^−3^		
				Selenocompound metabolism	2.75 × 10^−5^		
				Sulfur metabolism	1.42 × 10^−12^		
				Sulfur relay	9.24 × 10^−3^		
				Transporters	1.53 × 10^−11^		
10	62	NS	8.36 × 10^−21^	Prokaryotic defense system	2.24 × 10^−2^	NS	
11	55	NS	5.65 × 10^−30^	Starch and sucrose metabolism	6.06 × 10^−6^	GH5	9.18 × 10^−3^
						GH9	1.02 × 10^−4^
						GH45	1.43 × 10^−2^
12	45	6.31 × 10^−03^	4.48 × 10^−10^	NS		GH16	7.20 × 10^−3^
13	44	5.55 × 10^−16^	1.96 × 10^−31^	Chaperones and folding catalysts	7.28 × 10^−3^	NS	
14	38	NS	NS	NS		NS	
15	29	2.41 × 10^−07^	6.78 × 10^−10^	Carbohydrate metabolism	7.48 × 10^−4^	CBM35	3.32 × 10^−9^
						GH43	9.59 × 10^−9^
16	27	2.47 × 10^−18^	3.37 × 10^−13^	Carbohydrate metabolism	1.87 × 10^−2^	CBM6	1.03 × 10^−9^
						CE6	6.30 × 10^−5^
				Pentose and glucuronate interconversions	7.42 × 10^−3^	GH10	1.98 × 10^−4^
						GH30	2.76 × 10^−5^
						PL1	1.99 × 10^−6^
17	21	3.47 × 10^−14^	3.71 × 10^−13^	Galactose metabolism	6.87 × 10^−3^	CBM6	3.50 × 10^−20^
				Glycerolipid metabolism	3.98 × 10^−2^	GH27	4.71 10^−2^
				Glycosphingolipid biosynthesis	1.35 × 10^−2^	GH43	9.91 × 10^−10^
				Other glycan degradation	6.45 × 10^−4^	GH95	4.71 × 10^−2^
				Sphingolipid metabolism	6.45 × 10^−4^	GH141	4.71 × 10^−2^

Values for growth rate, substrate, KEGG Category, and CAZy category are *P* values, after adjusting for multiple comparisons (hypergeometric test, Holm’s correction [33])

*NS* not significant

### Genes affected by growth rate in *F. succinogenes* S85

Of the 639 genes affected by growth rate, 267 (41.78%) exhibited increased relative expression as growth rate increased and 372 (58.22%) showed increased relative expression as growth rate decreased (Table 3). Eight gene clusters were enriched for genes affected by growth rate: clusters 4, 7, 9, 12, 13, 15, 16, and 17 (Table 2). In cluster 9, expression of 61 out of the 78 total genes in this cluster (78.21%) were affected by growth rate and all 61 of these genes showed increased relative expression as growth rate increased. Cluster 9 was also significantly enriched in genes annotated to the following KEGG categories: ABC transporters, chloroalkane and chloroalkene degradation, cysteine and methionine metabolism, nitrogen metabolism, quorum sensing, selenocompound metabolism, sulfur metabolism, sulfur relay system, and transporters (Table 2). Gene clusters 13, 15, 16, and 17 had a combined total of 103 genes whose expression was affected by growth rate, which was 85.12% of all of the genes placed in these 4 clusters. Moreover, all 103 of these genes exhibited increased relative expression during slower rates of growth. Furthermore, these gene clusters were enriched in several KEGG categories: cluster 13 was enriched in genes annotated as chaperones and folding catalysts; cluster 15 was enriched in genes for carbohydrate metabolism; cluster 16 was enriched in genes for carbohydrate metabolism and pentose and glucuronate interconversions; cluster 17 was enriched in genes for galactose metabolism, glycerolipid metabolism, glycosphingolipid biosynthesis, other glycan degradation, and sphingolipid metabolism. Several of these gene clusters were also enriched in genes annotated to multiple CAZy families: cluster 15 was enriched in genes for CBM35 and GH43; cluster 16 was enriched in genes for CBM6, CE6, GH10, GH30, and PL1; cluster 17 was enriched in genes for CBM6, GH141, GH27, GH43, and GH95. Several of the CAZy families enriched in gene clusters 15, 16 and 17 were also significantly enriched among all genes affected by growth rate (CBM6, GH10, GH43, and PL1) compared to the S85 transcriptome as a whole (Table 4).

**Table 3:** Total genes affected by growth rate and substrate

Pair-wise comparison		Total genes
Growth rate		639
Faster rate	Slower rate	639
267	372	
Substrate		483
Cellulose (adherent)	Glucose	226
112	114	
Cellulose (adherent)	Cellobiose	164
78	86	
Cellulose (adherent)	Cellulose (planktonic)	184
0	184	
Cellulose (planktonic)	Glucose	276
227	49	
Cellulose (planktonic)	Cellobiose	228
205	23	
Cellobiose	Glucose	110
44	66	

Pair-wise comparison results are the number of genes up-regulated under a given condition for each specific comparison (FDR < 0.01 & LFC > 1.5)

**Table 4:** KEGG categories and CAZy families affected by growth rate and substrate

Effect	KEGG category	*P*_adj_*	CAZyme family	*P*_adj_*
Growth rate	Chloroalkane and chloroalkene degradation	5.58 × 10^−2^	CBM6	1.75 × 10^−10^
	Cysteine and methionine metabolism	9.44 × 10^−2^	GH10	1.34 × 10^−2^
	Sulfur metabolism	4.94 × 10^−2^	GH43	1.62^−4^
			PL1	1.34^−2^
Substrate	Sulfur metabolism	2.83 × 10^−3^	CBM35	2.53^−3^
			CBM6	9.59^−15^
			CE6	6.49^−2^
			GH9	2.07^−2^
			GH10	3.78^−2^
			GH43	3.53^−7^
			PL1	2.45^−3^

* Hypergeometric test, Holm’s correction (30)

### Genes affected by substrate in *F. succinogenes* S85

Of the 483 total genes affected by substrate, 226 differed in expression between cellulose-adherent populations and those growing on glucose; 164 were differentially expressed between cellulose-adherent populations and those growing on cellobiose; 184 were differentially expressed between cellulose-adherent and cellulose-planktonic cells; 276 were differentially expressed between cellulose-planktonic cells and those growing on glucose; 228 were differentially expressed between cellulose-planktonic cells and those growing on cellobiose; 110 differed in expression between populations growing on cellobiose and glucose (Table 3). All 184 genes differentially expressed between cellulose-adherent and cellulose-planktonic populations demonstrated elevated expression in cellulose-planktonic cells, relative to cellulose-adherent cells. The 483 total genes affected by substrate were enriched for the KEGG category sulfur metabolism compared to the entire S85 transcriptome (*P*_adj_ = 0.03, Holm’s correction [33]) (Table 4). Cellulose-adherent populations tended to have the highest expression [regularized-log2 transformed sequence count (*r*log)] of genes in the KEGG category sulfur metabolism (mean *r*log = 8.28 ± 1.35), followed by cellulose-planktonic cells (mean *r*log = 8.15 ± 1.31), those growing on cellobiose (mean *r*log = 8.03 ± 1.52), and finally, glucose fed cells (mean *r*log = 7.65 ± 1.48). Genes showing an effect of substrate on their expression were also enriched for the following CAZy families: CBM35, CBM6, CE6, GH10, GH43, and GH9 (Table 4).

Evidence for significant enrichment in genes showing an effect of carbon substrate on expression was observed for 10 of the 17 total gene clusters: clusters 4, 7, 9, 10, 11, 12, 13, 15, 16 and 17 (Table 2). Cluster 11 contained 55 genes, and included 47 whose expression was significantly affected by substrate. Genes in cluster 11 generally showed increased expression in cellulose-planktonic populations (mean *r*log = 8.95 ± 2.74) and cellulose-adherent populations (mean *r*log = 8.73 ± 2.90) versus those growing on cellobiose (mean *r*log = 7.88 ± 2.78) or glucose (mean *r*log = 7.68 ± 2.68). Cluster 11 was also significantly enriched for genes in CAZy families GH5, GH9 and GH45, as well as the KEGG category starch and sucrose metabolism. A high percentage of genes in clusters 13 and 17, 68.18% and 90.45%, respectively, showed elevated expression in cellulose-planktonic cells relative to the cellulose-adherent population. KEGG categories enriched in these clusters include: chaperones and folding catalysts, galactose metabolism, glycerolipid metabolism, glycosphingolipid biosynthesis, other glycan degradation, and sphingolipid metabolism (Table 2).

Two adjacent genes, Fisuc_0384 and Fisuc_0385, are noteworthy for their extremely high relative expression in cellulose-adherent and cellulose-planktonic populations relative to S85 populations growing on either soluble sugar (Additional file 1: Table S4). An LFC > 5.5 was observed for both genes during growth on cellulose, regardless of condition, relative to growth on either glucose or cellobiose. Fisuc_0384 and Fisuc_0385 are predicted to encode an uncharacterized protein involved in the regulation of septum location-like protein and a Mg chelatase, subunit ChlI, respectively.

### Expression of cellulases and hemicellulases by *F. succinogenes* S85

A total of 208 CAZy-annotated modules, encoded by 172 genes, were identified in the S85 transcriptome (Additional file 1: Table S5). All of the CAZy-annotated modules identified in the S85 genome were represented in the S85 transcriptome under at least one condition examined here. The CAZy-annotated gene exhibiting the highest mean gene expression across all samples was Fisuc_3111 (CBM11-GH51), the locus for Cel51A (also known as endoglucanase 2/endoglucanase F). In addition to being one of the most highly expressed genes in the transcriptome (> 99th percentile), Fisuc_3111 had a relatively low coefficient of variation (CV) for expression (2.12% across all samples), and was placed in cluster 1 by hierarchical clustering of all genes (Additional file 1: Table S2). Other CAZy genes with high (> 90th percentile) and relatively stable (< 5% CV) expression were: Fisuc_1802 (GH8), Fisuc_0393 (GH9), Fisuc_2900 (GH94, cellodextrin phosphorylase), Fisuc_1530 (GH18), Fisuc_2097 (GT35, glycogen phosphorylase), Fisuc_1224 (GH5), Fisuc_3049 (GH2), Fisuc_1219 (GH8), Fisuc_0668 (GH57), Fisuc_1932 (GH13), and Fisuc_2988 (GH23) (Additional file 1: Table S2).

**Fig. 3:**
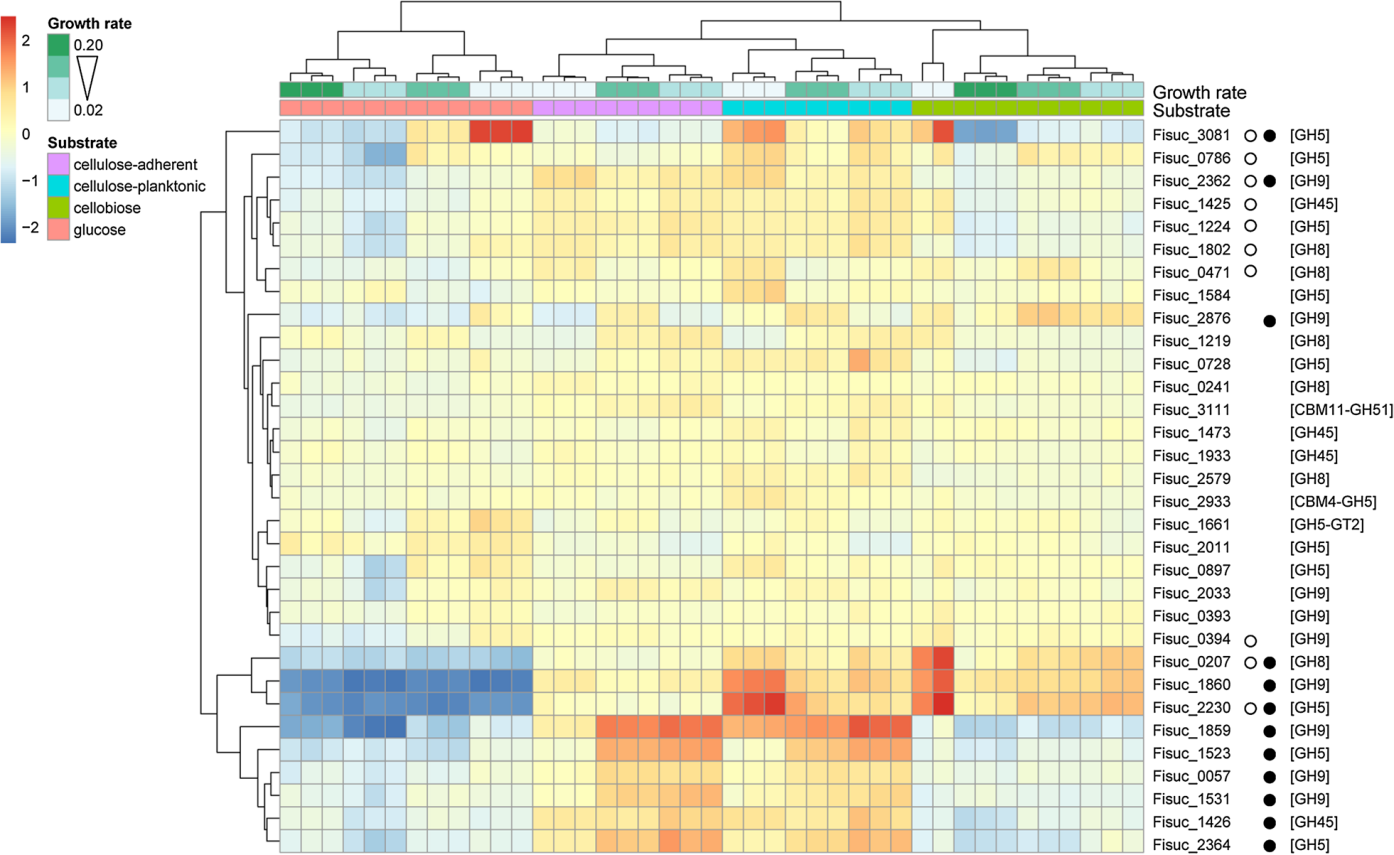
Expression of genes encoding cellulases. The heatmap shows the deviation from the mean *r*log expression across samples for cellulase-encoding genes in the CAZy families GH5, GH8, GH9, GH45, and GH51. Genes are arranged vertically according to the results of the hierarchical clustering. Samples are arranged horizontally according to the results of the hierarchical clustering, and colored according to substrate (purple = cellulose-adherent, blue = cellulose-planktonic, green = cellobiose, red = glucose) and growth rate (slow = light green and fast = dark green). The heatmap is colored according to log2 fold change from the mean for a gene in a given sample, with the intensity of red (positive change) or blue (negative change) indicating the magnitude of the effect. Open circles to the right of the locus ID indicate an effect of growth rate on gene expression, while solid circles indicate an effect of carbon substrate on expression. CAZy families for the genes are in brackets

A clustered heatmap showing deviations from the mean *r*log expression across the samples for genes (*n* = 32) annotated to the major CAZy cellulase families in the S85 genome (GH5, GH8, GH9, GH45, and GH51) is shown in Fig. 3. A significant effect of growth rate on expression was observed for 10 of the 32 cellulase genes (31.25%). All 10 of these cellulases demonstrated decreased relative expression as growth rate increased, after controlling for the effect of substrate. A significant effect of substrate type on expression was observed for 12 of the 32 cellulase genes (37.5%). Nine of the 12 fell into two clusters at the very bottom of the cellulase heatmap (Fig. 3). Cellulase genes affected by substrate generally exhibited the lowest levels of expression during growth on glucose. Two other distinct patterns of expression were observed among these cellulase genes. One pattern consisted of elevated expression during growth on cellulose, irrespective of attachment. The other was characterized by increased expression during growth on cellobiose, as well as in cellulose-adherent and cellulose-planktonic cells. Hierarchical clustering of all S85 genes based on similar gene expression patterns identified cluster 11 as being enriched for cellulase families GH5, GH9 and GH45 (Table 2).

**Fig. 4:**
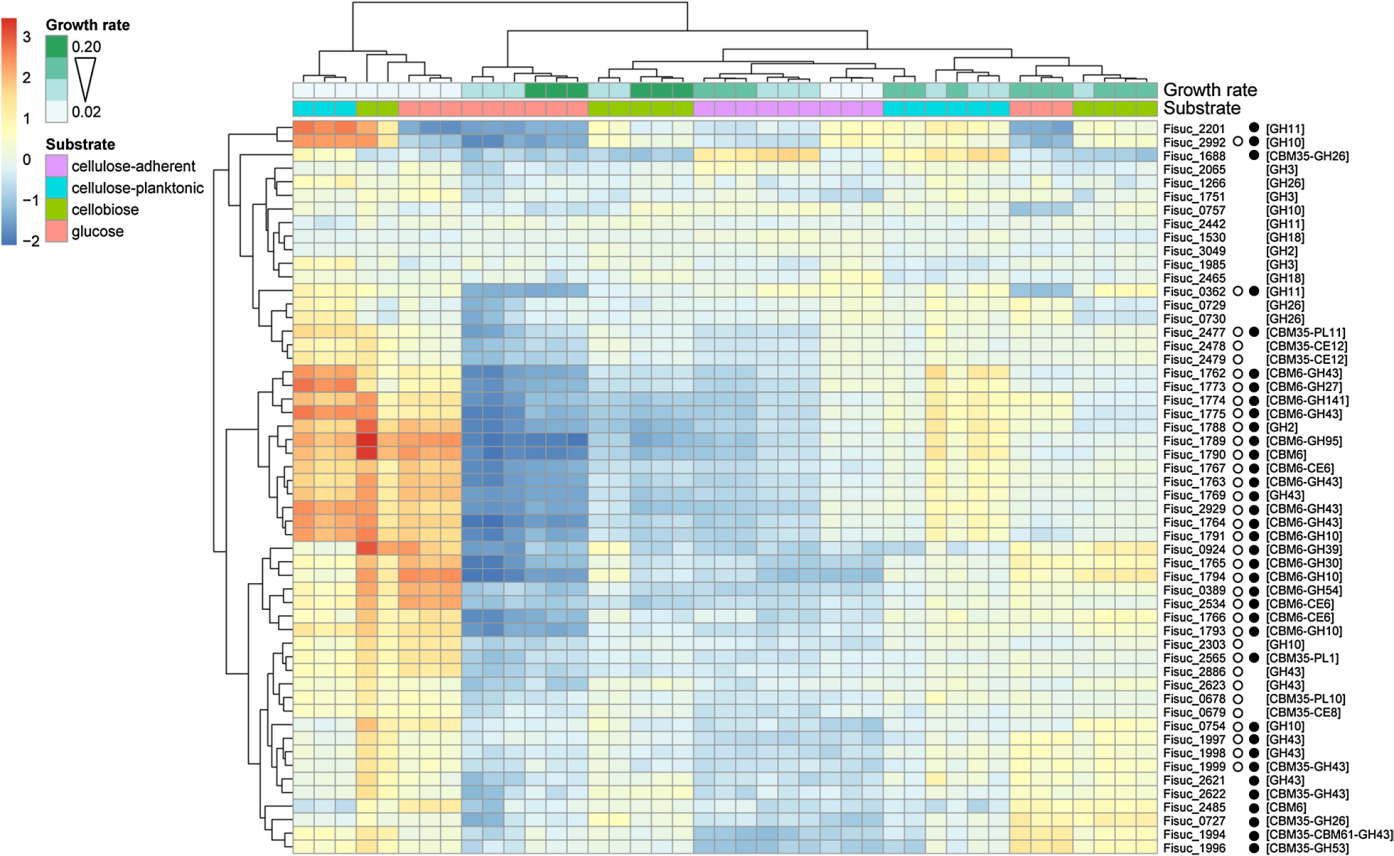
Expression of genes encoding hemicellulases. The heatmap shows the deviation from the mean *r*log expression across samples for genes encoding hemicellulases in the CAZy families CBM6, CBM35, GH2, GH3, GH10, GH11, GH18, GH26, and GH43. Genes are arranged vertically according to the results of the hierarchical clustering. Samples are arranged horizontally according to the results of the hierarchical clustering, and colored according to substrate (purple = cellulose-adherent, blue = cellulose-planktonic, green = cellobiose, red = glucose) and growth rate (slow = light green and fast = dark green). The heatmap is colored according to log2 fold change from the mean for a gene in a given sample, with the intensity of red (positive change) or blue (negative change) indicating the magnitude of the effect. Open circles to the right of the locus ID indicate an effect of growth rate on gene expression, while solid circles indicate an effect of carbon substrate on expression. CAZy families for the genes are in brackets

A clustered heatmap showing deviations from the mean *r*log expression across the samples for genes (*n* = 54) annotated to the major hemicellulase-related carbohydrate-binding module and glycoside hydrolase CAZy families in the S85 genome (CBM6, CBM35, GH2, GH3, GH10, GH11, GH18, GH26, GH43) is shown in Fig. 4. Three major patterns of gene expression were identified in the hemicellulase heatmap (Fig. 4), two of which varied considerably with substrate type and growth rate. One of the two variable expression patterns, consisting of elevated expression at the slowest growth rate (*D* ~ 0.02 h^−1^) in glucose, cellobiose, and especially cellulose-planktonic populations, was observed for many hemicellulases containing a CBM6 domain. The other was characterized by elevated relative expression during growth on soluble sugars, even at moderate growth rates, and relatively less expression evident in cellulose-planktonic populations. Most of the hemicellulase genes that possess a CBM35 domain exhibited this pattern of expression, as did the remainder of those encoding enzymes with a CBM6 domain.

Overall, a significant effect of growth rate on expression was detected for 35 of the 54 hemicellulases (64.81%). The expression of hemicellulase genes generally decreased as growth rate increased, regardless of substrate. Three CAZy families implicated in hemicellulose breakdown by S85 (CBM6, GH10 and GH43) were significantly enriched among the complete set of genes determined to be affected by growth rate (Table 4). Among the hemicellulase-related CAZy genes, a clear majority, 36 of 53 (67.92%), exhibited evidence of a significant effect of substrate type on their expression. Most demonstrated elevated expression in cells growing on soluble sugars relative to cellulose-adherent cells, 18/36 for glucose versus adherent, 29/36 for cellobiose versus adherent, and 21/36 for cellulose-planktonic versus adherent. Genes encoding for CBM35, CBM6, CE6, GH10, and GH43 modules, which are implicated in the breakdown of hemicellulose by S85, were also significantly enriched among all S85 genes affected by substrate type (Table 4). Hierarchical clustering of the expression profiles of all genes identified three clusters that were significantly enriched in hemicellulase-related CAZy families (Table 2). Cluster 15 was enriched in genes annotated to encode for CBM35 and GH43; cluster 16 was enriched in genes annotated to encode for CBM6, CE6 and GH10; and cluster 17 was enriched in the families CBM6 and GH43, as well as several other CAZy families with less understood target substrates in this bacterium.

## Discussion

In this study, we examined gene expression by *F. succinogenes* S85 at steady state in continuous culture on several carbon substrates over a range of different growth rates. The dilution rates used spanned almost the complete range for growth of this strain on cellulose (*µ*_max_ = 0.076 h^−1^ [34]). For soluble substrates, the maximum dilution rate was ~ 40% of the maximum growth rate measured for batch cultures [35], but was still somewhat faster than the minimum liquid-phase dilution rate within the rumen. Our data suggest that growth rate had a larger overall effect on the total S85 transcriptome than did any particular carbon substrate. This is supported by the results of PCA comparing the overall transcriptomes as well as hierarchical clustering of the samples. A total of 639 genes were identified as being affected by growth rate. Most of these genes showed increased relative expression during slower rates of growth compared to cultures growing at a faster rate, after controlling for the effect of substrate. Many of these genes are annotated as being involved in glycan degradation and are elaborated upon below. These results find some analogy to gene expression patterns in *Clostridium thermocellum* (phylum Firmicutes), one of the most intensively studied cellulolytic bacteria. In *C. thermocellum*, the total number of genes affected by growth rate exceeded the number affected by substrate type [36]. Moreover, two of the major genes associated with cellulose degradation, namely *cipA*, the cellulosomal scaffoldin, and *celS*, the major exoglucanase, display markedly higher expression at lower growth rates in cellobiose-limited cultures [37].

Of the genes that showed the opposite pattern of expression (i.e., increased relative expression at faster growth rates), many are annotated to functional categories related to sulfur metabolism. It is unclear why genes involved in sulfur metabolism are induced under any of the culture conditions examined in this study, as the use of cysteine as a reducing agent in the media, which is readily utilized as a source of sulfur by S85, should ensure that the concentration of sulfur always exceeds cellular requirements. *F. succinogenes* has been demonstrated to readily utilize l-cysteine as a source of sulfur to meet cellular requirements [38].

A large group of genes with elevated expression in strain S85 during slow growth, relative to higher growth rates, are annotated to CAZy families believed to be responsible for hemicellulose hydrolysis by S85. These same genes were also among those whose expression was most affected by carbon substrate. A notable characteristic of the Fibrobacteres is their inability to utilize the constituents of hemicellulose (other than hexoses) for growth, despite readily hydrolyzing this class of polysaccharides [39, 40]. This observation has led to the hypothesis that these bacteria primarily utilize these enzymes only as a means of gaining access to cellulose, which is typically embedded in a matrix of hemicellulose, pectin, and lignin, and that the process may be mediated through the production of outer membrane vesicles [22, 24]. Tight control over the genes coding for S85 hemicellulases is consistent with this hypothesis, as these enzymes are apparently only required under very specific circumstances. A slow growth rate may signal to the cell that its preferred substrate, cellulose, is not readily available and that the production of hemicellulases may assist in gaining access to it. Our results also suggest that cells of S85 are able to distinguish between slow rates of growth while attached to cellulose versus slow growth on glucose or cellobiose because cellulose-adherent was the only substrate condition examined where genes for hemicellulases did not show elevated expression at the slowest growth rate examined (*D* ~ 0.02 h^−1^). One hypothesis is that the presence of glucose or cellobiose in combination with a low rate of growth signals to the cell that cellulose is near, but not readily available. Under these conditions, the induction of genes for hemicellulases would be beneficial. Moreover, the particular sensitivity of the S85 cellulase system to feedback inhibition [41] potentially indicates that even during slow growth on cellulose, cellobiose and glucose are consumed immediately as they are produced, ensuring that the signal for hemicellulase induction remains low.

It is well established that cellulose degradation by *Fibrobacter* likely involves many enzymes working synergistically, but the conditions under which they are expressed are less well understood. Our observations support a model with several distinct patterns of expression among cellulase genes by strain S85. The CAZy-annotated gene showing the highest levels of expression across growth rates and carbon substrates was Fisuc_3111, the gene encoding Cel51A (also known as endoglucanase 2/endoglucanase F). This enzyme was one of the first endoglucanases to be purified from S85, in addition to being one of the first major cellulose-binding proteins identified in this strain [42, 43]. Cel51A is also the only GH51 family protein in the S85 genome [22]. Our results suggest that this enzyme is expressed constitutively, based on a low coefficient of variation in its expression across all conditions. This is consistent with proteomic data which identified Cel51A in the outer membrane of S85 regardless of whether it was cultured on glucose, acid-swollen cellulose, or microcrystalline cellulose [44]. Additionally, Cel51A has been implicated in outer membrane blebbing and vesicle formation because the protein is largely cell associated until encountering cellulose, at which point it becomes concentrated in areas of blebbing and membrane disruption as it translocates to the cellulose surface, taking pieces of the outer membrane with it [43, 45]. Constitutive expression of the gene encoding for Cel51A would ensure that plenty of mRNA for producing more of this cellulase is always available to replace the protein as it is sheared out of the membrane upon contact with cellulose. Along with Fisuc_3111, our data suggest that 14 of the 32 43.75%) cellulase genes in the S85 genome are constitutively expressed. These genes represent all families of cellulases possessed by S85: GH5, GH8, GH9, GH45, and GH51. Constitutive expression of at least a subset of cellulases explains why S85 exhibits no lag in growth when transferred to cellulose after being grown on soluble sugars [29].

Two additional patterns of expression were observed among the cellulase-encoding genes in S85. One pattern, represented by Fisuc_0207, Fisuc_1860 (encoding Cel9C/CelD), and Fisuc_2230 (encoding Cel5G/Cel3), consisted of very low relative expression on glucose and increased expression in both cellulose subpopulations as well as in cells growing on cellobiose. These three cellulase-encoding genes were most highly expressed in cellulose-planktonic and cellobiose populations, particularly at the slowest growth rate. These results suggest that cellobiose, either when fed directly or produced from cellulose hydrolysis, is involved in stimulating the expression of these genes. Another major pattern of expression observed among the S85 cellulase-encoding genes included at least six genes, one of which was Fisuc_1859, the gene for Cel9B (also known as endoglucanase 1/endoglucanase E), that displayed elevated expression in cellulose-adherent and cellulose-planktonic populations, but not during growth on cellobiose. Moreover, expression of these genes tended to increase along with growth rate during growth on cellulose, which was atypical for glycoside hydrolases in this study, and also in *C. thermocellum* [36, 37]. Cel9B, endoglucanase 1, has been shown to be released during growth by S85 and to contribute a large fraction of the extracellular endoglucanase activity produced by this bacterium [42, 45]. Our results confirm previous reports that expression of this enzyme is regulated by substrate, viz. repressed by cellobiose, which is also the major product produced by this enzyme [45]. Tight control over the expression of *cel9B* likely helps to ensure that this endoglucanase is efficiently delivered to its substrate and that the substrate is already in close proximity to the cell such that the cellobiose produced can be rapidly consumed before it is lost to competitors.

In addition to hydrolytic enzymes, proteins that promote cellular attachment to cellulose are critically important for S85 because adherence is essential for efficient growth on cellulose [19]. S85 proteins containing a fibro-slime domain have been implicated in attachment to cellulose by these bacteria [23], and the S85 genome has ten genes predicted to encode for the domain [22]. Our data show that several of these genes are among the most highly expressed genes in the S85 transcriptome, with three in the top 98th percentile across all conditions: Fisuc_1979, Fisuc_1474, and Fisuc_0377. Previously, we reported that 8 of the 10 fibro-slime genes were upregulated during growth on cellulose relative to growth on glucose [46]. However, the data generated in this study suggest that a majority of fibro-slime genes are expressed constitutively. Evidence of an effect on expression by growth rate and carbon source was observed for only two fibro-slime genes, for each variable. The most likely explanation for the discrepancy is that because our previous work examined populations undergoing log-phase growth in batch cultures, differences in growth rate between the substrates confounded the interpretation of the differences in gene expression. These results emphasize the importance of controlling for growth rate when evaluating gene expression, as shown previously for *C. thermocellum* [36, 37].

The S85 population in cellulose-limited continuous cultures is heterogeneous, consisting primarily of cells that are adherent to the cellulose surface and a much smaller fraction of cells that are planktonic (i.e., not adherent). Microscopic observations of planktonic cells undergoing cell division support the conclusion that both subpopulations are engaging in cellular growth, but likely utilizing different substrates [47]. As has been reported previously, we observed a low concentration of soluble sugar (~ 1 mM), consisting primarily of glucose (as indicated by an average degree of polymerization of 1.5), present in steady-state cellulose cultures, further demonstrating that substrate is indeed available to feed planktonic cells under these conditions [34]. Therefore, we fractionated these two subpopulations prior to RNA extraction to investigate their gene expression separately. Our results identified clear differences in gene expression between the two populations. Patterns of gene expression characteristic of cellulose-planktonic populations exhibited some commonalities with both cellulose-adherent populations and those growing on soluble sugars. Several cellulase-encoding genes displayed elevated expression in both cellulose-adherent and cellulose-planktonic populations, but not in populations growing on either soluble sugar. However, genes encoding hemicellulases, which were minimally expressed in cellulose-adherent populations, showed elevated expression during slow growth in cellulose-planktonic populations as well as during slow growth on glucose and cellobiose. A likely explanation for these observations is that cellulose-planktonic populations are heterogenous in nature, consisting of recently released daughter cells of the cellulose-adherent population that exhibit gene expression patterns resembling that of their mother cells, as well as cells that have undergone several cycles of division growing on the soluble sugars available in the liquid phase of the culture. Our observations that cellulose-adherent and -planktonic populations display differences in gene expression support the work of Dumitrache et al. with the thermophilic cellulose degrader *C. thermocellum* [48]. Those workers used a novel reactor design to permit separate collection of adherent and planktonic cells. However, those experiments were conducted in batch culture and the two subpopulations appeared to grow at markedly different rates (undefined for adherent cells, and *µ*_max_ of up to 0.82 h^−1^ for planktonic cells, six times faster than this strain’s known *µ*_max_ on cellulose). This confounding of adherence status by growth rate prevents direct comparison of gene expression in these subpopulations.

The observation that continuous cultures on all three substrates (cellulose, glucose and cellobiose) contained ~ 1 mM (glucose equivalent) of residual soluble sugars is consistent with the relatively poor affinity of this strain for soluble sugars (Ks ~ 0.5 mM for both cellulose and cellobiose [35]). The average degree of polymerization of these sugars (based on a ratio of total sugars/reducing sugars) was ~ 1.4 across the three substrates fed, indicating that glucose was the most abundant of the residual sugars. The identities of the other sugars could include cellobiose (either remaining from cellobiose feeding, or produced from glucose by reversal of cellobiose phosphorylase), and small amounts of longer cellodextrins produced in the same manner [47]. These residual sugars could also potentially include maltose and maltodextrins produced from turnover of glycogen, as these carbohydrates have been detected in extracellular media during batch culture growth of S85 on soluble sugars [14]. The intriguing potential role of glycogen turnover in S85 cellular metabolism is supported by our observations that two annotated glycogen synthases (Fisuc_1515 and Fisuc_2067) and a glycogen phosphorylase (Fisuc_2097) were highly expressed, independent of growth rate or substrate type. Further research is needed to identify and quantify each of these potential soluble sugars, and to elucidate the interplay between the catabolism of cellulose and its hydrolytic products on the one hand, and glycogen synthesis and turnover on the other. Regardless, our finding that S85 displayed differential expression of over 100 genes when grown on glucose versus cellobiose suggests that these two sugars have distinct roles in the metabolic regulation of this bacterium.

## Conclusion

This study provides the most detailed view of the *F. succinogenes* S85 transcriptome to date, and is the first systematic assessment of the effect of growth rate and carbon substrate on gene expression in this important succinate-producing rumen bacterium. We have shown for the first time tight control over global expression of genes encoding hemicellulases in these bacteria as well as multiple distinct patterns of expression for cellulase-encoding genes. This work reinforces the importance of controlling for growth rate when evaluating effects on gene expression. We have also demonstrated differences in gene expression between adherent and planktonic populations in a bacterial culture growing on an insoluble substrate, even after controlling for growth rate, a variable that is often overlooked in studies of anaerobic fiber-degraders. Future work focused on how the patterns of gene expression described here relate to the protein levels in S85 is warranted.

## Methods

### Bacterial strain, culture conditions and sampling

The *Fibrobacter succinogenes* type strain, S85 (ATCC 19169), was used for all experiments in this study. The medium used for cultivation was a slightly modified version of the formula first described by Scott and Dehority [49]. The growth medium used here lacked acetic acid and casein hydrolysate, but was, otherwise, effectively identical. The exact contents of the medium are listed in Additional file 1: Table S6. Carbon-limited continuous cultures were conducted at 39 °C (pH = 6.8) using a bioreactor (working volume = 762 mL) system capable of regulated delivery of an insoluble substrate as a segmented slurry, as described previously [50]. Carbon sources tested included glucose, cellobiose, and microcrystalline cellulose (Sigmacell 20, Sigma-Aldrich, St. Louis, MO). Sigmacell 20 was utilized because its small particle size allowed homogeneous suspension during aggressive mixing in the reservoir and reactor [50]. The soluble sugars were included in the medium at 2 g L^−1^, while the concentration of cellulose used was 3.6 g L^−1^. The higher concentration of cellulose was used because, unlike for the soluble sugars, a substantial fraction of the cellulose is not consumed during continuous culture of *F. succinogenes* S85 [34]. Soluble substrates were also delivered as a segmented slurry to allow the use of similar rotor speeds in the peristaltic delivery pump. The amount of cellulose used was determined from previous empirical observations with the goal of normalizing overall substrate consumption, cell growth, and product formation. Sampling of continuous cultures growing on either glucose or cellobiose was performed at four targeted dilution rates: *D* = 0.018, 0.047, 0.068, and 0.20 h^−1^. Sampling of continuous cultures growing on crystalline cellulose was performed at three targeted dilution rates: *D* = 0.018, 0.047, and 0.068 h^−1^. Samples corresponding with the fastest dilution rate evaluated for the soluble sugars (*D* = 0.20 h^−1^) could not be collected from cellulose-limited continuous cultures because wash-out occurs for this organism during growth on crystalline cellulose at dilution rates greater than 0.08 h^−1^ [34]. Three samples were collected at 5–20-h intervals for each carbon source and dilution rate combination at steady state (> 3 reactor volumes, equivalent to > 97% volume turnover) for transcriptomic analysis as well as quantification of fermentation products, residual soluble sugars and, in the case of the cellulose-limited continuous cultures, residual cellulose in the reactor. Consistent with chemostat theory, growth rate was assumed to equal the dilution rate for steady-state continuous cultures.

### Substrate and product analysis

Supernatants collected from continuous cultures at steady state were centrifuged for 10 min at 12,000×*g* and analyzed for total soluble sugar content using a phenol/sulfuric acid method [51], with glucose as the standard. Reducing sugars were measured by the dinitrosalicylic acid method, using glucose as the standard [52]. Supernatants were also analyzed for fermentation acids and alcohols by high-performance liquid chromatography (HPLC) after treatment with calcium hydroxide and cupric sulfate, with identical chromatographic conditions to those reported previously [50]. Two technical replicates were analyzed for each biological replicate, and the results were averaged. Normality and homoscedasticity of the data were assessed and linear regression and analysis of variance (ANOVA) performed to determine the effect of growth rate, carbon source, and potential interaction between the two explanatory variables on the production of the major fermentation products, succinate and acetate. Cellulose concentration in the reservoir and residual cellulose in the reactor were determined gravimetrically from ~ 20 g samples (weighed to 0.001 g) after treatment with boiling neutral detergent [53] to remove adherent bacterial cells, and subtracted from the initial concentration to calculate the grams of cellulose consumed per liter. Protein was determined using the Bradford method, with lysozyme as standard [54], after digesting cell pellets in 0.1 N KOH for 20 min at 70 °C. Mean values are reported with accompanying standard deviations.

### RNA extraction, library prep and sequencing

Total RNA was extracted as described previously [55]. Cells from 10 mL of glucose-limited or cellobiose-limited continuous cultures were harvested via centrifugation (12,000×*g*, 10 min, 5 °C) and resuspended in 1 mL RNA extraction buffer (50 mM sodium acetate, 10 mM EDTA, pH = 5.1). Cells from 10 mL of cellulose-limited continuous cultures were first separated into cellulose-adherent and cellulose-planktonic fractions by low speed centrifugation at 1000×*g* for 5 min. The supernatant, containing the cellulose-planktonic population, was then collected and processed separately. Cells from both the cellulose-adherent and cellulose-planktonic populations were subsequently recovered by centrifugation (12,000×*g*, 10 min, 5 °C) and resuspended in 1 mL RNA extraction buffer, as above. The cell suspension was combined with 0.5 g of 0.1 mm zirconium beads, 700 µL equilibrated phenol (pH < 5.0) and 50 µL 20% SDS. The sample was subjected to bead-beating for 2 min, followed by incubation at 60° C for 10 min, followed by additional bead-beating for 2 min. The organic and aqueous phases were separated via centrifugation and the aqueous phase extracted with 500 µL equilibrated phenol. Four additional extractions were performed, two with phenol:chloroform (50:50) followed by two with chloroform only. The RNA was precipitated with 3 M Na acetate and isopropanol, and resuspended in RNase-free H_2_O. Residual DNA contamination was removed using TURBO DNA-free (Life technologies, Carlsbad, CA). Total RNA was quantified using the RNA HS assay kit and Qubit^®^ Fluorometer (Invitrogen, Carlsbad, CA) prior to shipment to the DOE Joint Genome Institute (Walnut Creek, CA) for library preparation and sequencing. rRNA was removed from 100 ng of total RNA using the Ribo-Zero™ rRNA Removal Kit (Epicentre, Madison, WI). Stranded cDNA libraries were generated using the Illumina Truseq Stranded RNA LT kit (Illumina, San Diego, CA). The rRNA-depleted RNA was fragmented and reversed transcribed using random hexamers and SSII (Invitrogen) followed by second-strand synthesis. The fragmented cDNA was treated with end-pair, A-tailing, adapter ligation, and 10 cycles of PCR. qPCR was used to determine the concentration of the libraries. Libraries were sequenced on the Illumina Hiseq-2500 using the 2 × 100 bp paired end run mode.

### Preprocessing, alignment and counting of sequencing reads

Raw reads were filtered and trimmed using BBDuk (https://sourceforge.net/projects/bbmap/). Specifically, raw reads were evaluated for artifact sequence by kmer matching (kmer = 25), allowing 1 mismatch, and detected artifacts trimmed from the 3′ end of the reads. RNA spike-in reads, PhiX reads and reads containing any Ns were removed. Quality trimming was performed using the phred trimming method set at Q10, and any reads under 45 bases in length were removed. The resulting reads from each library were aligned to the *F. succinogenes* S85 reference genome (IMG Taxonoid 646311927) using BBMap (https://sourceforge.net/projects/bbmap/) with only unique mappings allowed. If a read mapped to more than one location it was discarded. featureCounts [56] was used to generate raw gene counts. The extent of correlation between biological samples was evaluated from the raw gene counts using Pearson’s correlation. One cellobiose sample from the lowest dilution rate (*D* = 0.016), library BBZPZ (Additional file 1: Table S7), was clearly deviant from the other biological replicates sampled for this substrate and growth rate and was, therefore, excluded from the transcriptomic analysis (see Additional file 2: Figures S1 and S2).

### Differential expression

Raw gene counts were imported into R (https://www.r-project.org/) and tested for differential expression using the “DESeq2” package (http://www.bioconductor.org/packages/release/bioc/html/DESeq2.html) [57]. A DESeqDataSet object was generated for differential expression analysis from the complete data set with the design formula: expression ~ growth rate + substrate type, with growth rate treated as a continuous variable and substrate type treated as a categorical variable. Empirically determined dilution rates were used as values for growth rate. The likelihood ratio test was used to determine which genes showed evidence of a statistically significant effect of either carbon source or growth rate on expression by comparing the full model, containing both explanatory variables, to a reduced model without the variable of interest. Independent hypothesis weighting was performed using the R Bioconductor package “IHW” [58] to optimize the power of the statistical tests. The false discovery rate (FDR) was controlled < 0.01 using the method of Benjamini and Hochberg [59]. A log2 fold change (LFC) > 1.5 was used as the criterion for biological significance.

### Ordination and clustering

Raw gene counts were transformed using the regularized-logarithm transformation (*r*log), log base 2, prior to exploratory analyses to ensure that the gene expression data were normalized across samples and approximately homoscedastic [57]. Mean *r*log values are presented along with ± 1 standard deviation. Principal components analysis (PCA) of sample–sample distances was performed by passing the *r*log-transformed data to the *plotPCA* function of “DESeq2” [57]. Clustering of samples was performed by calculating the Euclidean distance between the samples from the *r*log-transformed data and passing the resulting distance matrix to the *hclust* function from base R, which by default performs complete-linkage hierarchical clustering. Clustering of genes was performed by first calculating the deviation from the mean in each sample for all genes by subtracting a gene’s average expression across all samples from its expression in a single sample. Euclidean distances between the genes were then calculated from the deviations and used as input for clustering using *hclust*. Gene clusters were extracted from the dendrogram using the *cutreeDynamic* function from the R package “dynamicTreeCut” [60] with a minimum cluster size of 20. Clustered heatmaps of deviations for select genes were generated using *pheatmap* from the R package “pheatmap”.

### Gene annotation

Kyoto Encyclopedia of Genes and Genomes (KEGG) pathway annotations were determined using the BlastKOALA online annotation tool (http://www.kegg.jp/blastkoala/) for KOID number assignment [61]. Carbohydrate-active enzyme database (CAZy) annotation was performed using HMMER, v3.2 [62] and the dbCAN database, v6 [63]. CAZy annotations with *e*-value > 1 × 10^−18^ and coverage < 0.35 were discarded, as recommended for bacteria (http://csbl.bmb.uga.edu/dbCAN/). The hypergeometric test (equivalent to a one-sided Fisher’s exact test) was used to determine significant enrichment of a functional category among a particular subset of genes of interest. *P* values were adjusted to control the family-wise error rate across multiple comparisons using the base R function *p.adjust*, which uses Holm’s correction method by default [33].

### Sequence accession numbers

Raw Illumina sequencing reads have been deposited in the National Center for Biotechnology Information’s Short Read Archive (SRA) database under accessions PRJNA402842–PRJNA402853 and PRJNA402860–PRJNA402889. More detailed information pertaining to the individual libraries and their corresponding SRA accessions can be found in Additional file 1: Table S7.

## Additional files

**Additional file 1: Table S1.** Cellular protein measurements. **Table S2.** Normalized expression - summary statistics for all genes and samples. **Table S3.** Genes affected by growth rate. **Table S4.** Genes affected by substrate. **Table S5.** CAZy gene - summary statistics. **Table S6.** Media composition. **Table S7.** Library data.

**Additional file 2: Figure S1.** Principal components analysis of transcriptomes (all samples). **Figure S2.** Hierarchical clustering of transcriptomes (all samples).
