# Supplementary material for: A global analysis of gene expression in Fibrobacter succinogenes S85 grown on cellulose and soluble sugars at different growth rates
Source: Biotechnol Biofuels. 2018 Oct 27;11:295. doi: 10.1186/s13068-018-1290-x (PMC6204037; doi:10.1186/s13068-018-1290-x)
Supplement: Supplementary file 2 — Additional file 2: Figure S1. Principal components analysis of transcriptomes (all samples). Figure S2. Hierarchical clustering of transcriptomes (all samples). [file 13068_2018_1290_MOESM2_ESM.pdf]

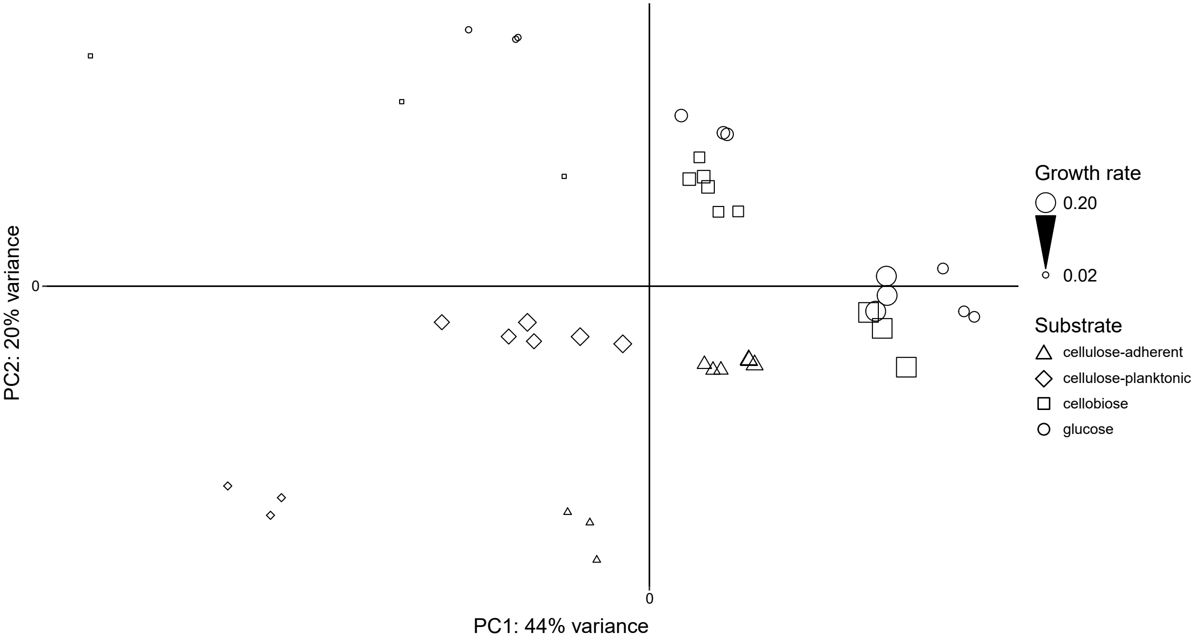

Figure S1. Principal components analysis of transcriptomes. The scatter plot shows the transcriptomes plotted according to the first two dimensions of principal components analysis. Cellulose-adherent samples are plotted as open triangles, cellulose-planktonic samples are plotted as open diamonds, cellobiose samples are plotted as open squares, and glucose samples are plotted as open circles. The sizes of the shapes increase as a function of growth rate.

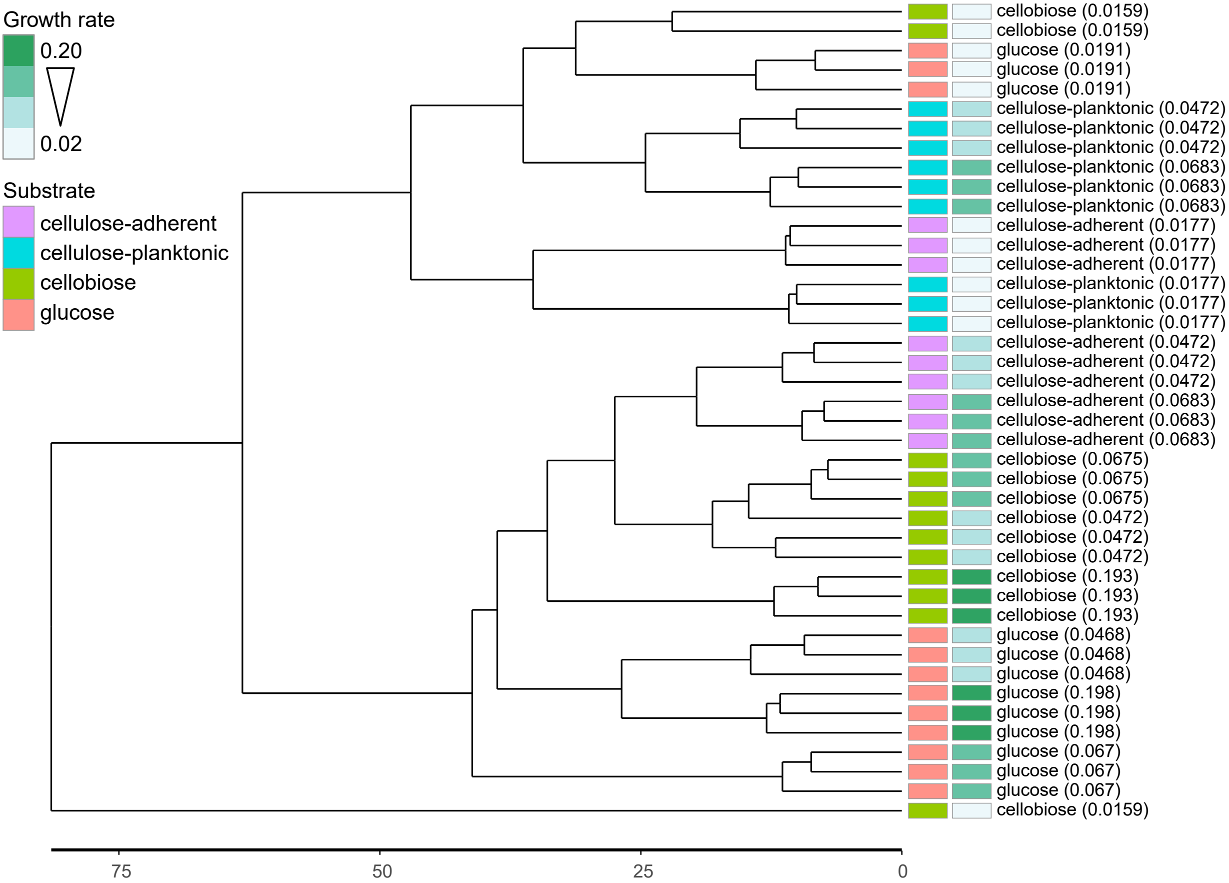

Figure S2. Hierarchical clustering of transcriptomes. The dendrogram shows the results of complete-linkage hierarchical clustering of the transcriptomes based on Euclidean distance. Samples are marked by colored according to substrate as follows: purple = cellulose-adherent, blue = cellulose-planktonic, green = cellobiose, red = glucose. Samples are separately colored according to growth rate from light (slow) to dark green (fast). Samples are labeled according to substrate type followed by empirically determined growth rates in parentheses.
